# The genome sequence of a satellite fly, *Leucophora obtusa *(Zetterstedt, 1837)

**DOI:** 10.12688/wellcomeopenres.19920.1

**Published:** 2023-09-04

**Authors:** Steven Falk, Rachel Lennon, University of Oxford and Wytham Woods Genome Acquisition Lab, Darwin Tree of Life Barcoding collective, Wellcome Sanger Institute Tree of Life programme, Wellcome Sanger Institute Scientific Operations: DNA Pipelines collective, Tree of Life Core Informatics collective, Darwin Tree of Life Consortium

**Affiliations:** 1 Independent researcher, Kenilworth, England, UK; 2 School of Biodiversity, One Health and Veterinary Medicine, University of Glasgow, Glasgow, Scotland, UK

**Keywords:** Leucophora obtusa, a satellite fly, genome sequence, chromosomal, Diptera

## Abstract

We present a genome assembly from an individual female *Leucophora obtusa* (a satellite fly; Arthropoda; Insecta; Diptera; Anthomyiidae). The genome sequence is 1,289.8 megabases in span. Most of the assembly is scaffolded into 6 chromosomal pseudomolecules, including the X sex chromosome. The mitochondrial genome has also been assembled and is 18.72 kilobases in length.

## Species taxonomy

Eukaryota; Metazoa; Eumetazoa; Bilateria; Protostomia; Ecdysozoa; Panarthropoda; Arthropoda; Mandibulata; Pancrustacea; Hexapoda; Insecta; Dicondylia; Pterygota; Neoptera; Endopterygota; Diptera; Brachycera; Muscomorpha; Eremoneura; Cyclorrhapha; Schizophora; Calyptratae; Muscoidea; Anthomyiidae; Anthomyiinae; *Leucophora*; *Leucophora obtusa* (Zetterstedt, 1838) (NCBI:txid2588525).

## Background

*Leucophora* is a genus of root-maggot flies in the family Anthomyiidae, which has over 60 described species, with *Leucophora obtusa* being recorded most often. *Leucophora obtusa* is found across Japan, Europe and North America ( GBIF Secretariat, 2022; Suwa, 1974). *L. obtusa* parasitise the larvae of *Andrena* bees, and can often be found near their nests ( Huckett, 1940; Polidori *et al.*, 2005). The common name “satellite fly” comes from the observed behaviour of the female fly hovering or “orbiting” around bee nests. Females are seen to shadow the host bee back to its burrow where she will oviposit in the tumulus of the nest entrance, and *L. obtusa* larvae are parasitic on the host’s brood ( Michener & Rettenmeyer, 1956; Polidori *et al.*, 2005).

This species is notoriously difficult to distinguish from other flies in the *Leucophora* genus and has often been misidentified throughout the literature ( Collin, 2009; Huckett, 1940). The genus itself can be distinguished by broad parafacials at the base of the antennae and are yellow basally and dark brownish apically ( Suwa, 1974). *L. obtusa* is seen as a particularly hairy species of this genus, with a hairy thorax, abdomen, and legs ( Huckett, 1940). It possesses long, erect hairs on the abdominal sternites and lateral margins of the scutellum, and the mid tibia and hind femur also have long fine ventral setae ( Suwa, 1974).

We hope that this novel, chromosomally complete genome sequence, developed as part of the Darwin Tree of Life Project, can be of benefit to continued understanding of the biology of *L. obtusa.* This project is a collaborative effort to sequence all named eukaryotic species in the Atlantic Archipelago of Britain and Ireland.

## Genome sequence report

**Figure 1.**
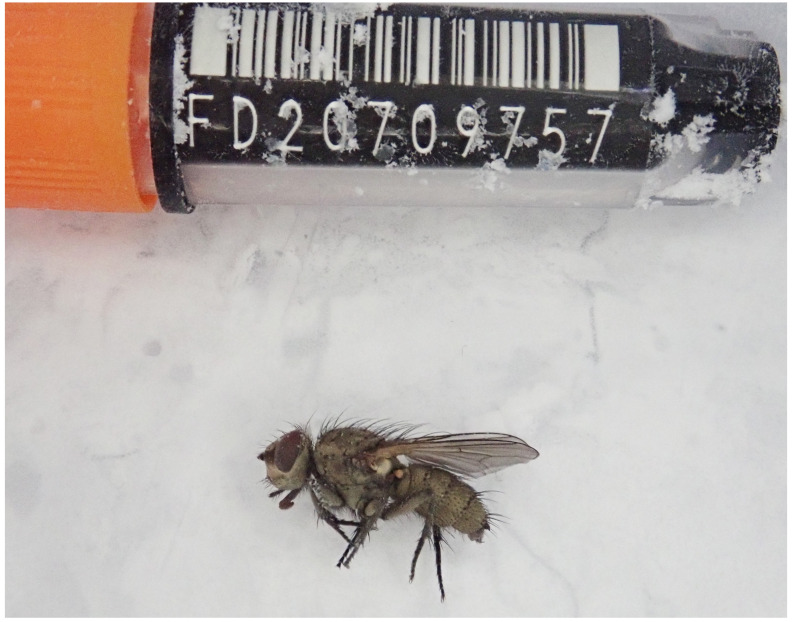
Photograph of the *Leucophora obtusa* (idLeuObtu2) specimen used for genome sequencing.

The genome was sequenced from one female *Leucophora obtusa* ( Figure 1) collected from Wytham Woods, Oxfordshire, UK (51.76, –1.34). A total of 33-fold coverage in Pacific Biosciences single-molecule HiFi long reads was generated. Primary assembly contigs were scaffolded with chromosome conformation Hi-C data. Manual assembly curation corrected 72 missing joins or mis-joins and removed 3 haplotypic duplications, reducing the scaffold number by 17.21%, and increasing the scaffold N50 by 1.22%.

**Figure 2.**
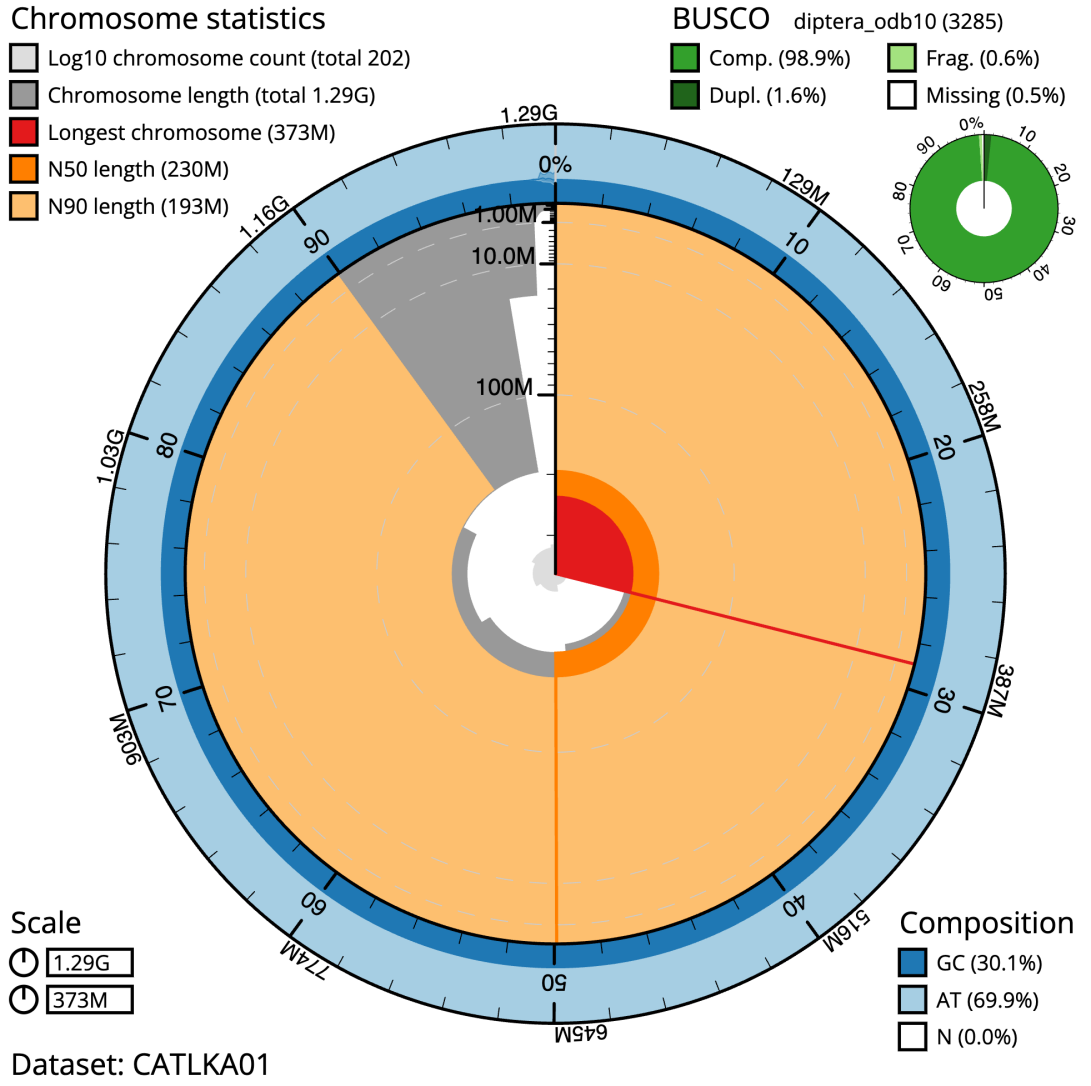
Genome assembly of *Leucophora obtusa*, idLeuObtu2.1: metrics. The BlobToolKit Snailplot shows N50 metrics and BUSCO gene completeness. The main plot is divided into 1,000 size-ordered bins around the circumference with each bin representing 0.1% of the 1,289,857,006 bp assembly. The distribution of scaffold lengths is shown in dark grey with the plot radius scaled to the longest scaffold present in the assembly (373,316,775 bp, shown in red). Orange and pale-orange arcs show the N50 and N90 scaffold lengths (230,154,751 and 193,475,527 bp), respectively. The pale grey spiral shows the cumulative scaffold count on a log scale with white scale lines showing successive orders of magnitude. The blue and pale-blue area around the outside of the plot shows the distribution of GC, AT and N percentages in the same bins as the inner plot. A summary of complete, fragmented, duplicated and missing BUSCO genes in the diptera_odb10 set is shown in the top right. An interactive version of this figure is available at https://blobtoolkit.genomehubs.org/view/idLeuObtu2.1/dataset/CATLKA01/snail.

The final assembly has a total length of 1,289.8 Mb in 201 sequence scaffolds with a scaffold N50 of 230.2 Mb ( Table 1). Most (99.17%) of the assembly sequence was assigned to 6 chromosomal-level scaffolds. Chromosome-scale scaffolds confirmed by the Hi-C data are named in order of size ( Figure 2– Figure 5; Table 2). The exact order and orientation of the scaffolds in the repetitive centromeres is unknown. Since idLeuObtu2 is a female XX sample lacking sequence data from the heterogametic species, the X chromosome remains unidentified. This is because, in Diptera, homology is unreliable for sex chromosome identification due to frequent turnover of these chromosomes ( Vicoso & Bachtrog, 2015). While not fully phased, the assembly deposited is of one haplotype. Contigs corresponding to the second haplotype have also been deposited. The mitochondrial genome was also assembled and can be found as a contig within the multifasta file of the genome submission.

**Table 1.** Genome data for *Leucophora obtusa*, idLeuObtu2.1.

Project accession data		
Assembly identifier	idLeuObtu2.1	
Species	*Leucophora obtusa*	
Specimen	idLeuObtu2	
NCBI taxonomy ID	2588525	
BioProject	PRJEB57107	
BioSample ID	SAMEA10166765	
Isolate information	idLeuObtu2, female: whole organism (DNA sequencing and Hi-C data) idLeuObtu3, female: whole organism, (RNA sequencing)	
Assembly metrics *		*Benchmark*
Consensus quality (QV)	60.8	*≥ 50*
*k*-mer completeness	100%	*≥ 95%*
BUSCO **	C:98.9%[S:97.3%,D:1.6%], F:0.6%,M:0.5%,n:3,285	*C ≥ 95%*
Percentage of assembly mapped to chromosomes	99.17%	*≥ 95%*
Sex chromosomes	Not identified	*localised homologous pairs*
Organelles	Mitochondrial genome assembled	*complete single alleles*
Raw data accessions		
PacificBiosciences SEQUEL II	ERR10439748, ERR10439749	
Hi-C Illumina	ERR10446383	
PolyA RNA-Seq Illumina	ERR10890707	
Genome assembly		
Assembly accession	GCA_949987735.1	
*Accession of alternate * *haplotype*	GCA_949987745.1	
Span (Mb)	1,289.8	
Number of contigs	814	
Contig N50 length (Mb)	4.3	
Number of scaffolds	201	
Scaffold N50 length (Mb)	230.2	
Longest scaffold (Mb)	373.3	

* Assembly metric benchmarks are adapted from column VGP-2020 of “Table 1: Proposed standards and metrics for defining genome assembly quality” from ( Rhie *et al.*, 2021). ** BUSCO scores based on the diptera_odb10 BUSCO set using v5.3.2. C = complete [S = single copy, D = duplicated], F = fragmented, M = missing, n = number of orthologues in comparison. A full set of BUSCO scores is available at https://blobtoolkit.genomehubs.org/view/idLeuObtu2.1/dataset/CATLKA01/busco.

**Figure 3.**
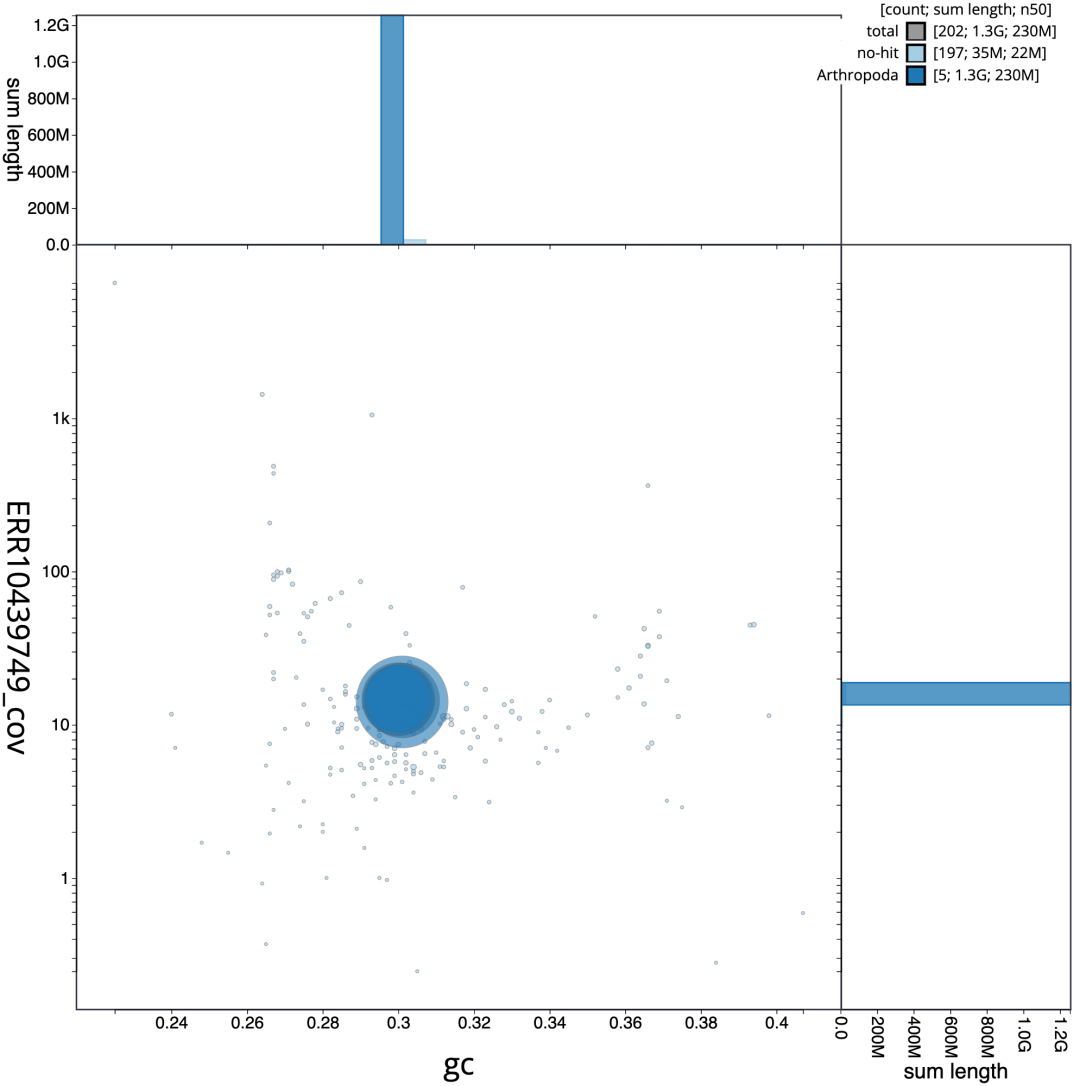
Genome assembly of *Leucophora obtusa*, idLeuObtu2.1: BlobToolKit GC-coverage plot. Scaffolds are coloured by phylum. Circles are sized in proportion to scaffold length. Histograms show the distribution of scaffold length sum along each axis. An interactive version of this figure is available at https://blobtoolkit.genomehubs.org/view/idLeuObtu2.1/dataset/CATLKA01/blob.

**Figure 4.**
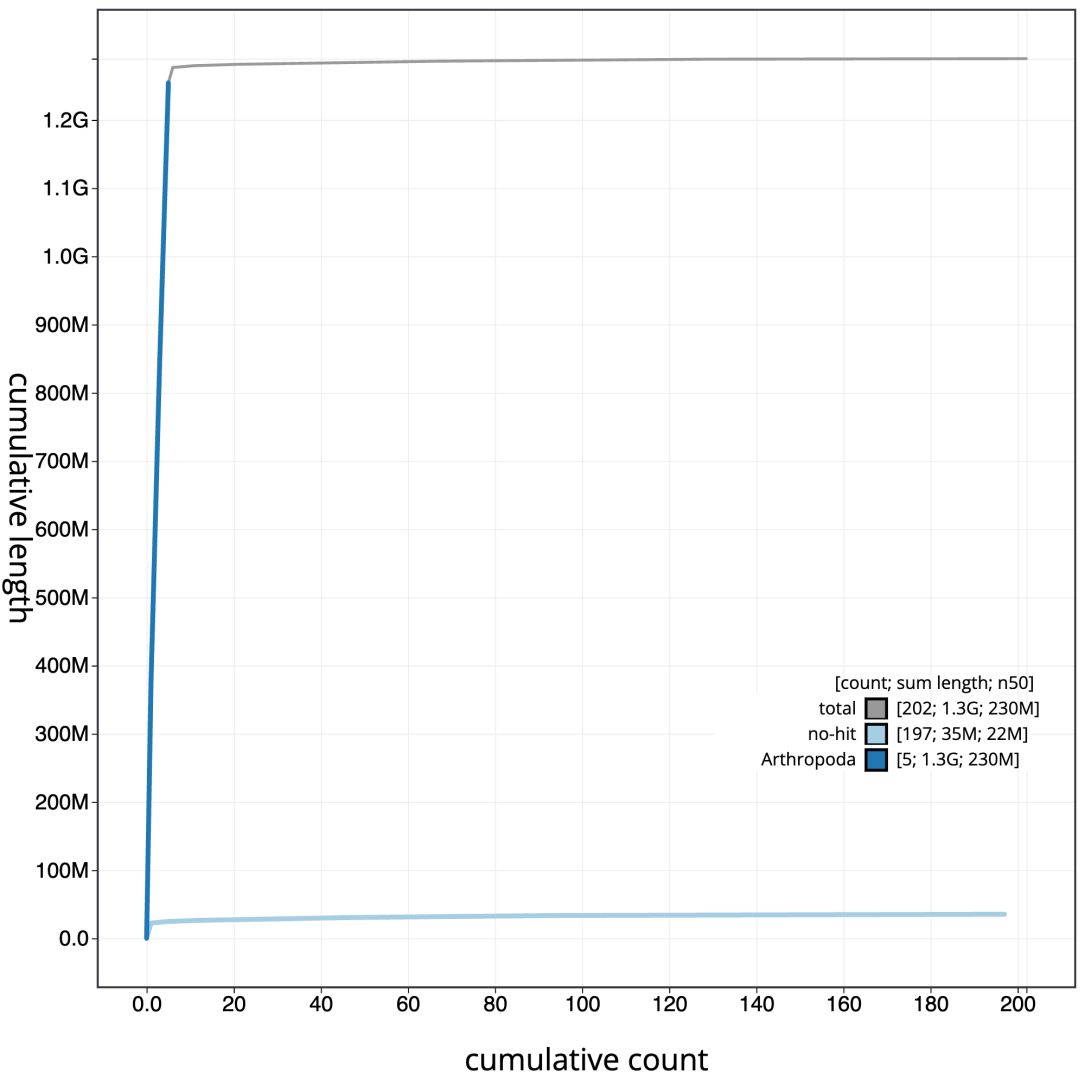
Genome assembly of *Leucophora obtusa*, idLeuObtu2.1: BlobToolKit cumulative sequence plot. The grey line shows cumulative length for all scaffolds. Coloured lines show cumulative lengths of scaffolds assigned to each phylum using the buscogenes taxrule. An interactive version of this figure is available at https://blobtoolkit.genomehubs.org/view/idLeuObtu2.1/dataset/CATLKA01/cumulative.

**Figure 5.**
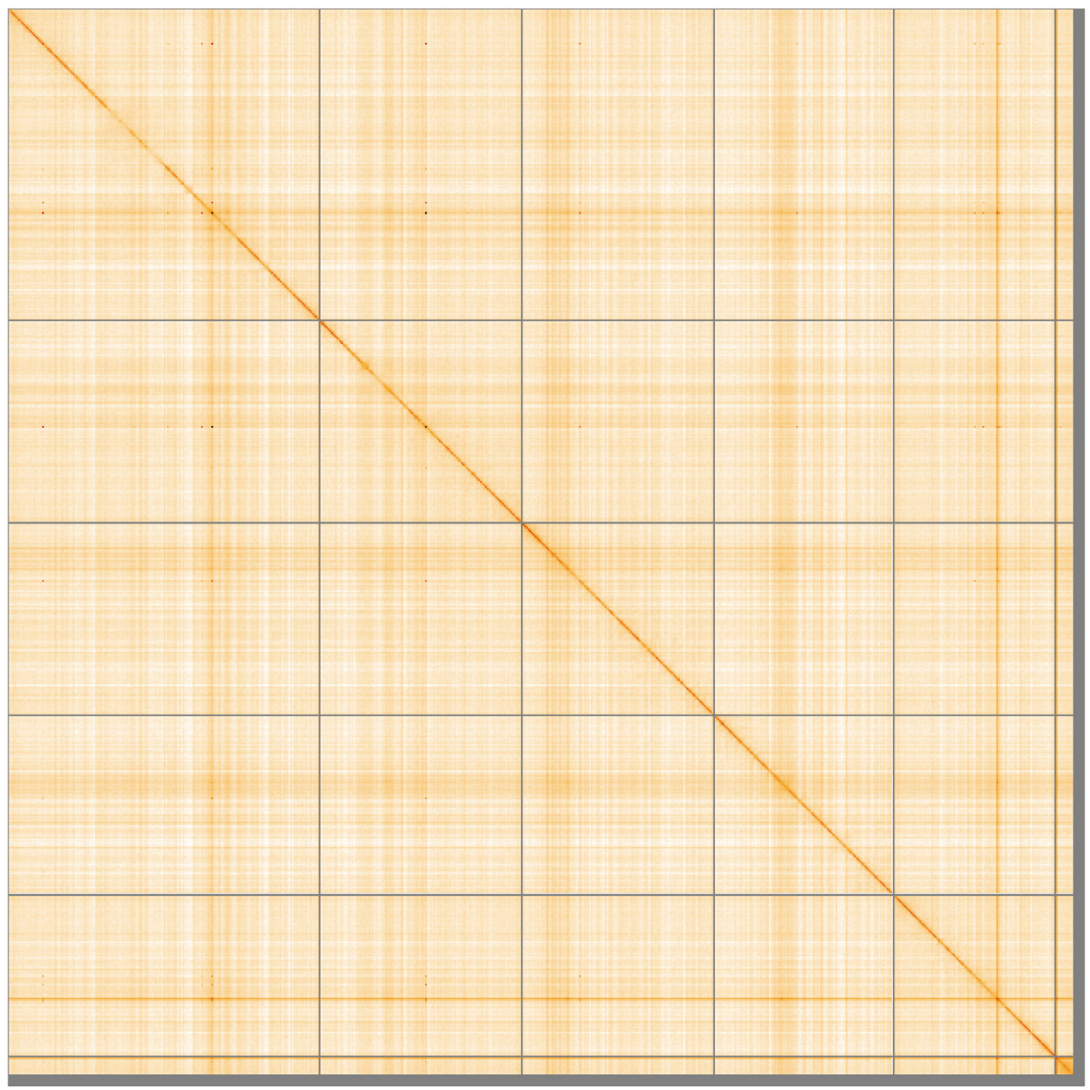
Genome assembly of *Leucophora obtusa*, idLeuObtu2.1: Hi-C contact map of the idLeuObtu2.1 assembly, visualised using HiGlass. Chromosomes are shown in order of size from left to right and top to bottom. An interactive version of this figure may be viewed at https://genome-note-higlass.tol.sanger.ac.uk/l/?d=DbS5c8XOSS6dl4NdSeCc7A.

**Table 2.** Chromosomal pseudomolecules in the genome assembly of *Leucophora obtusa*, idLeuObtu2.

INSDC accession	Chromosome	Length (Mb)	GC%
OX465295.1	1	373.32	30.0
OX465296.1	2	242.24	30.0
OX465297.1	3	230.15	30.0
OX465298.1	4	215.25	30.0
OX465299.1	5	193.48	30.0
OX465300.1	6	22.42	30.0
OX465301.1	MT	0.02	22.5

The estimated Quality Value (QV) of the final assembly is 60.8 with *k*-mer completeness of 100%, and the assembly has a BUSCO v5.3.2 completeness of 98.9% (single = 97.3%, duplicated = 1.6%), using the diptera_odb10 reference set ( *n* = 3,285).

Metadata for specimens, spectral estimates, sequencing runs, contaminants and pre-curation assembly statistics can be found at https://links.tol.sanger.ac.uk/species/2588525.

## Methods

### Sample acquisition and nucleic acid extraction

*Leucophora obtusa* specimens were collected from Wytham Woods, Oxfordshire (biological vice-county Berkshire), UK (latitude 51.76, longitude –1.34) on 2021-04-23 by netting. The specimens were collected and identified by Steven Falk (independent researcher), and were then preserved on dry ice. The specimen used for genome sequencing and Hi-C data was a female with specimen ID Ox001285 (ToLID idLeuObtu2), while the specimen used for RNA sequencing has specimen ID Ox001305 (ToLID idLeuObtu3).

DNA was extracted at the Tree of Life laboratory, Wellcome Sanger Institute (WSI). The idLeuObtu2 sample was weighed and dissected on dry ice with tissue set aside for Hi-C sequencing. Tissue from the whole organism was disrupted using a Nippi Powermasher fitted with a BioMasher pestle. High molecular weight (HMW) DNA was extracted using the Qiagen MagAttract HMW DNA extraction kit. HMW DNA was sheared into an average fragment size of 12–20 kb in a Megaruptor 3 system with speed setting 30. Sheared DNA was purified by solid-phase reversible immobilisation using AMPure PB beads with a 1.8X ratio of beads to sample to remove the shorter fragments and concentrate the DNA sample. The concentration of the sheared and purified DNA was assessed using a Nanodrop spectrophotometer and Qubit Fluorometer and Qubit dsDNA High Sensitivity Assay kit. Fragment size distribution was evaluated by running the sample on the FemtoPulse system.

RNA was extracted from whole organism tissue of idLeuObtu3 in the Tree of Life Laboratory at the WSI using TRIzol, according to the manufacturer’s instructions. RNA was then eluted in 50 μl RNAse-free water and its concentration assessed using a Nanodrop spectrophotometer and Qubit Fluorometer using the Qubit RNA Broad-Range (BR) Assay kit. Analysis of the integrity of the RNA was done using Agilent RNA 6000 Pico Kit and Eukaryotic Total RNA assay.

### Sequencing

Pacific Biosciences HiFi circular consensus DNA sequencing libraries were constructed according to the manufacturers’ instructions. Poly(A) RNA-Seq libraries were constructed using the NEB Ultra II RNA Library Prep kit. DNA and RNA sequencing was performed by the Scientific Operations core at the WSI on Pacific Biosciences SEQUEL II (HiFi) and Illumina NovaSeq 6000, (RNA-Seq) instruments. Hi-C data were also generated from remaining tissue of idLeuObtu2 using the Arima2 kit and sequenced on the Illumina NovaSeq 6000 instrument.

### Genome assembly, curation and evaluation

Assembly was carried out with Hifiasm ( Cheng *et al.*, 2021) and haplotypic duplication was identified and removed with purge_dups ( Guan *et al.*, 2020). The assembly was then scaffolded with Hi-C data ( Rao *et al.*, 2014) using YaHS ( Zhou *et al.*, 2023). The assembly was checked for contamination and corrected as described previously ( Howe *et al.*, 2021). Manual curation was performed using HiGlass ( Kerpedjiev *et al.*, 2018) and Pretext ( Harry, 2022). The mitochondrial genome was assembled using MitoHiFi ( Uliano-Silva *et al.*, 2023), which runs MitoFinder ( Allio *et al.*, 2020) or MITOS ( Bernt *et al.*, 2013) and uses these annotations to select the final mitochondrial contig and to ensure the general quality of the sequence.

A Hi-C map for the final assembly was produced using bwa-mem2 ( Vasimuddin *et al.*, 2019) in the Cooler file format ( Abdennur & Mirny, 2020). To assess the assembly metrics, the *k*-mer completeness and QV consensus quality values were calculated in Merqury ( Rhie *et al.*, 2020). This work was done using Nextflow ( Di Tommaso *et al.*, 2017) DSL2 pipelines “sanger-tol/readmapping” ( Surana *et al.*, 2023a) and “sanger-tol/genomenote” ( Surana *et al.*, 2023b). The genome was analysed within the BlobToolKit environment ( Challis *et al.*, 2020) and BUSCO scores ( Manni *et al.*, 2021; Simão *et al.*, 2015) were calculated.

Table 3 contains a list of relevant software tool versions and sources.

**Table 3.** Software tools: versions and sources.

Software tool	Version	Source
BlobToolKit	4.0.7	https://github.com/blobtoolkit/blobtoolkit
BUSCO	5.3.2	https://gitlab.com/ezlab/busco
Hifiasm	0.16.1-r375	https://github.com/chhylp123/hifiasm
HiGlass	1.11.6	https://github.com/higlass/higlass
Merqury	MerquryFK	https://github.com/thegenemyers/MERQURY.FK
MitoHiFi	2	https://github.com/marcelauliano/MitoHiFi
PretextView	0.2	https://github.com/wtsi-hpag/PretextView
purge_dups	1.2.3	https://github.com/dfguan/purge_dups
sanger-tol/genomenote	v1.0	https://github.com/sanger-tol/genomenote
sanger-tol/readmapping	1.1.0	https://github.com/sanger-tol/readmapping/tree/1.1.0
YaHS	yahs-1.2a.2	https://github.com/c-zhou/yahs

### Wellcome Sanger Institute – Legal and Governance

The materials that have contributed to this genome note have been supplied by a Darwin Tree of Life Partner. The submission of materials by a Darwin Tree of Life Partner is subject to the **‘Darwin Tree of Life Project Sampling Code of Practice’**, which can be found in full on the Darwin Tree of Life website here. By agreeing with and signing up to the Sampling Code of Practice, the Darwin Tree of Life Partner agrees they will meet the legal and ethical requirements and standards set out within this document in respect of all samples acquired for, and supplied to, the Darwin Tree of Life Project. 

Further, the Wellcome Sanger Institute employs a process whereby due diligence is carried out proportionate to the nature of the materials themselves, and the circumstances under which they have been/are to be collected and provided for use. The purpose of this is to address and mitigate any potential legal and/or ethical implications of receipt and use of the materials as part of the research project, and to ensure that in doing so we align with best practice wherever possible. The overarching areas of consideration are:

• Ethical review of provenance and sourcing of the material

• Legality of collection, transfer and use (national and international) 

Each transfer of samples is further undertaken according to a Research Collaboration Agreement or Material Transfer Agreement entered into by the Darwin Tree of Life Partner, Genome Research Limited (operating as the Wellcome Sanger Institute), and in some circumstances other Darwin Tree of Life collaborators.

## Data Availability

European Nucleotide Archive: *Leucophora obtusa*. Accession number PRJEB57107; https://identifiers.org/ena.embl/PRJEB57107. ( Wellcome Sanger Institute, 2023) The genome sequence is released openly for reuse. The *Leucophora obtusa* genome sequencing initiative is part of the Darwin Tree of Life (DToL) project. All raw sequence data and the assembly have been deposited in INSDC databases. The genome will be annotated using available RNA-Seq data and presented through the Ensembl pipeline at the European Bioinformatics Institute. Raw data and assembly accession identifiers are reported in Table 1.

## Author information

Members of the University of Oxford and Wytham Woods Genome Acquisition Lab are listed here: https://doi.org/10.5281/zenodo.4789928.

Members of the Darwin Tree of Life Barcoding collective are listed here: https://doi.org/10.5281/zenodo.4893703.

Members of the Wellcome Sanger Institute Tree of Life programme are listed here: https://doi.org/10.5281/zenodo.4783585.

Members of Wellcome Sanger Institute Scientific Operations: DNA Pipelines collective are listed here: https://doi.org/10.5281/zenodo.4790455.

Members of the Tree of Life Core Informatics collective are listed here: https://doi.org/10.5281/zenodo.5013541.

Members of the Darwin Tree of Life Consortium are listed here: https://doi.org/10.5281/zenodo.4783558.

## References

[ref-1] Abdennur N Mirny LA : Cooler: Scalable storage for Hi-C data and other genomically labeled arrays. *Bioinformatics.* 2020;36(1):311–316. 10.1093/bioinformatics/btz540 31290943 PMC8205516

[ref-2] Allio R Schomaker-Bastos A Romiguier J : MitoFinder: Efficient automated large‐scale extraction of mitogenomic data in target enrichment phylogenomics. *Mol Ecol Resour.* 2020;20(4):892–905. 10.1111/1755-0998.13160 32243090 PMC7497042

[ref-4] Bernt M Donath A Jühling F : MITOS: Improved *de novo* metazoan mitochondrial genome annotation. *Mol Phylogenet Evol.* 2013;69(2):313–319. 10.1016/j.ympev.2012.08.023 22982435

[ref-6] Challis R Richards E Rajan J : BlobToolKit - interactive quality assessment of genome assemblies. *G3 (Bethesda).* 2020;10(4):1361–1374. 10.1534/g3.119.400908 32071071 PMC7144090

[ref-7] Cheng H Concepcion GT Feng X : Haplotype-resolved *de novo* assembly using phased assembly graphs with hifiasm. *Nat Methods.* 2021;18(2):170–175. 10.1038/s41592-020-01056-5 33526886 PMC7961889

[ref-31] Collin JE : XII. A Contribution towards the knowledge of the Anthomyid genera *Hammomyia* and *Hylephila* of Rondani (Diptera). *Transactions of the Royal Entomological Society of London.* 2009;68(3–5):305–326. 10.1111/j.1365-2311.1921.tb00224.x

[ref-8] Di Tommaso P Chatzou M Floden EW : Nextflow enables reproducible computational workflows. *Nat Biotechnol.* 2017;35(4):316–319. 10.1038/nbt.3820 28398311

[ref-32] GBIF Secretariat: *Leucophora obtusa* (Zetterstedt, 1837). *GBIF Backbone Taxonomy.* Checklist dataset,2022; [Accessed 7 August 2023]. Reference Source

[ref-10] Guan D McCarthy SA Wood J : Identifying and removing haplotypic duplication in primary genome assemblies. *Bioinformatics.* 2020;36(9):2896–2898. 10.1093/bioinformatics/btaa025 31971576 PMC7203741

[ref-11] Harry E : PretextView (Paired REadTEXTure Viewer): A desktop application for viewing pretext contact maps. 2022; Accessed 19 October 2022. Reference Source

[ref-12] Howe K Chow W Collins J : Significantly improving the quality of genome assemblies through curation. *GigaScience.* Oxford University Press,2021;10(1): giaa153. 10.1093/gigascience/giaa153 33420778 PMC7794651

[ref-33] Huckett HC : The North American species of the genera *Leucophora* Robineau-Desvoidy and *Proboscimyia* Bigot (Muscidae, Diptera). *Journal of the New York Entomological Society.* 1940;48(4):335–365. Reference Source

[ref-13] Kerpedjiev P Abdennur N Lekschas F : HiGlass: web-based visual exploration and analysis of genome interaction maps. *Genome Biol. * 2018;19(1): 125. 10.1186/s13059-018-1486-1 30143029 PMC6109259

[ref-14] Manni M Berkeley MR Seppey M : BUSCO update: Novel and streamlined workflows along with broader and deeper phylogenetic coverage for scoring of eukaryotic, prokaryotic, and viral genomes. *Mol Biol Evol.* 2021;38(10):4647–4654. 10.1093/molbev/msab199 34320186 PMC8476166

[ref-34] Michener CD Rettenmeyer CW : The ethology of *Andrena erythronii* with comparative data on other species (Hymenoptera, Andrenidae).University of Kansas Science Bulletin,1956;37:645–684. Reference Source

[ref-35] Polidori C Scanni B Scamoni E : Satellite flies ( *Leucophora personata* Diptera: Anthomyiidae) and other dipteran parasites of the communal bee *Andrena agilissima* (Hymenoptera: Andrenidae) on the island of Elba, Italy. *Journal of Natural History.* 2005;39(29):2745–2758. 10.1080/00222930500114210

[ref-16] Rao SSP Huntley MH Durand NC : A 3D map of the human genome at kilobase resolution reveals principles of chromatin looping. *Cell.* 2014;159(7):1665–1680. 10.1016/j.cell.2014.11.021 25497547 PMC5635824

[ref-17] Rhie A McCarthy SA Fedrigo O : Towards complete and error-free genome assemblies of all vertebrate species. *Nature.* 2021;592(7856):737–746. 10.1038/s41586-021-03451-0 33911273 PMC8081667

[ref-18] Rhie A Walenz BP Koren S : Merqury: Reference-free quality, completeness, and phasing assessment for genome assemblies. *Genome Biol.* 2020;21(1): 245. 10.1186/s13059-020-02134-9 32928274 PMC7488777

[ref-19] Simão FA Waterhouse RM Ioannidis P : BUSCO: assessing genome assembly and annotation completeness with single-copy orthologs. *Bioinformatics.* 2015;31(19):3210–3212. 10.1093/bioinformatics/btv351 26059717

[ref-22] Surana P Muffato M Qi G : sanger-tol/readmapping: sanger-tol/readmapping v1.1.0 - Hebridean Black (1.1.0). *Zenodo.* 2023a; Accessed 21 July 2023. 10.5281/zenodo.7755665

[ref-23] Surana P Muffato M Sadasivan Baby C : sanger-tol/genomenote (v1.0.dev). *Zenodo.* 2023b; Accessed 21 July 2023. Reference Source

[ref-36] Suwa M : Anthomyiidae of Japan (Diptera).Insecta Matsumurana. New Series: Journal of the Faculty of Agriculture Hokkaido University, Series Entomology,1974;4:1–274. Reference Source

[ref-24] Uliano-Silva M Ferreira JGRN Krasheninnikova K : MitoHiFi: a python pipeline for mitochondrial genome assembly from PacBio high fidelity reads. *BMC Bioinformatics.* 2023;24(1): 288. 10.1186/s12859-023-05385-y 37464285 PMC10354987

[ref-25] Vasimuddin Md Misra S Li H : Efficient Architecture-Aware Acceleration of BWA-MEM for Multicore Systems. In: *2019 IEEE International Parallel and Distributed Processing Symposium(IPDPS).*IEEE,2019;314–324. 10.1109/IPDPS.2019.00041

[ref-38] Vicoso B Bachtrog D : Numerous Transitions of Sex Chromosomes in Diptera. *PLoS Biol.* 2015;13(4): e1002078. 10.1371/journal.pbio.1002078 25879221 PMC4400102

[ref-28] Wellcome Sanger Institute: The genome sequence of a satellite fly, *Leucophora obtusa* (Zetterstedt, 1837). European Nucleotide Archive.[dataset], accession number PRJEB57107,2023. 10.12688/wellcomeopenres.19920.1 PMC10811419 38283326

[ref-27] Zhou C McCarthy SA Durbin R : YaHS: yet another Hi-C scaffolding tool. *Bioinformatics.* 2023;39(1): btac808. 10.1093/bioinformatics/btac808 36525368 PMC9848053

