# Supplementary material for: The genome sequence of a satellite fly, Leucophora obtusa (Zetterstedt, 1837)
Source: Wellcome Open Res. 2023 Sep 4;8:392. [Version 1] doi: 10.12688/wellcomeopenres.19920.1 (PMC10811419; doi:10.12688/wellcomeopenres.19920.1)
Supplement: Peer review history

# Reviewer report: Reviewer response for version 1

**DOI:** 10.21956/wellcomeopenres.22059.r72151

**Published:** 2024-01-25

**Authors:** Markus Friedrich

**Affiliations:** 1 Department of Biological Sciences, Wayne State University, Detroit, Michigan, USA

**I think 1,289.8 Mb is exceptionally large for a dipteran genome. The authors should consider adding a statement or short discussion of genome sizes in related Diptera.**

**1.** **“We present a genome assembly from an individual female Leucophora obtusa (a satellite fly; Arthropoda; Insecta; Diptera; Anthomyiidae”**

**genome assembly FOR an individual**

**2.** **“behaviour of the female fly hovering or “orbiting” around bee nests.”**

**behavior of the female fly hovering OVER or “orbiting” around bee nests.**

**3.“The genus itself can be distinguished by broad parafacial at the base of the antennae and are yellow basally and dark brownish apically (Suwa, 1974).”**

**a. Define or replace “parafacial” with a less specialized term.**

**b. Split the sentence into two bc the noun of the second sentence is not clear.**

**4. “Since idLeuObtu2 is a female XX sample.”**

**Abbreviation idLeuObtu2 is introduced later in the Methods section. So, either introduce here or refer to “sequenced female individual.”**

**5.** **“The idLeuObtu2 sample was weighed and dissected on dry ice with tissue set aside for Hi-C sequencing.”**

**Which parts were removed, and which were subjected to DNA extraction?**

**And how does this relate to “Hi-C data were also generated from remaining tissue of idLeuObtu2 “**

**6.** **“TRIzol, according to the manufacturer’s instructions.”**

**Provide manufacturer.**

**7.** **“Illumina NovaSeq 6000, (RNA-Seq) instruments”**

**I think the comma after 6000 can be deleted.**

**8.“haplotypic duplication was identified.”**

**You removed 3 duplications, hence.**

**haplotypic duplications WERE identified.**

**9.“to select the final mitochondrial contig and to ensure the general quality of the sequence.”**

**Delete the “to” before “ensure.”**

Are sufficient details of methods and materials provided to allow replication by others?

Yes

Is the rationale for creating the dataset(s) clearly described?

Yes

Are the datasets clearly presented in a useable and accessible format?

Yes

Are the protocols appropriate and is the work technically sound?

Yes

Reviewer Expertise:

Comparative genomics of arthropods

I confirm that I have read this submission and believe that I have an appropriate level of expertise to confirm that it is of an acceptable scientific standard.

# Reviewer report: Reviewer response for version 1

**DOI:** 10.21956/wellcomeopenres.22059.r66575

**Published:** 2023-10-23

**Authors:** Yuqiang Xi

**Affiliations:** 1 State Key Laboratory of Wheat and Maize Crop Science/Henan International Laboratory for Green Pest Control, Department of Entomology, Henan Agricultural University, Zhengzhou, China

This study conduct genome sequencing using HGS approach. Most of the resulting data can be scaffolded into 6 chromosomal pseudomolecules. In addition, a 18.72 kilobases mitogenome was reconstructed from the genome sequencing data. Many new sequencing assembling software were employed in this study. The overall writing of the article is good. Although the raw data of the genome sequencing is already included in PRJEB57107, more detailed annotations regarding the genome (including the mitochondrial genome) should be publicly disclosed for future research.

 I suggested this article to be indexed.

Are sufficient details of methods and materials provided to allow replication by others?

Yes

Is the rationale for creating the dataset(s) clearly described?

Yes

Are the datasets clearly presented in a useable and accessible format?

Yes

Are the protocols appropriate and is the work technically sound?

Yes

Reviewer Expertise:

Insect systematics, molecular biology

I confirm that I have read this submission and believe that I have an appropriate level of expertise to confirm that it is of an acceptable scientific standard.
